# The Role of tRNA-Derived Small RNAs (tsRNAs) in Regulating Cell Death of Cardiovascular Diseases

**DOI:** 10.3390/biology14020218

**Published:** 2025-02-19

**Authors:** Jiaxu Guo, Xinzhe Chen, Jiahao Ren, Yunhong Wang, Kun Wang, Sumin Yang

**Affiliations:** 1 Department of Cardiovascular Surgery, Institute of Chronic Diseases, The Affiliated Hospital of Qingdao University, College of Medicine, Qingdao University, Qingdao 266021, China; gjxg123@163.com (J.G.); sdchenxinzhe@163.com (X.C.); r19862725253@163.com (J.R.); 2 State Key Laboratory of Cardiovascular Disease, Heart Failure Center, Fuwai Hospital, National Center for Cardiovascular Diseases, Chinese Academy of Medical Sciences, Peking Union Medical College, Beijing 100037, China; wangyunhong123@139.com

**Keywords:** cancer, cardiovascular disease, cell death, transfer RNA, tRNA-derived small RNA

## Abstract

Transfer RNA is a class of non-coding RNA that plays a role in amino acid translocation during protein synthesis. After specific modification, the cleaved fragment is called tRNA-derived small RNA. The advancement of bioinformatics technology has led to an increase in the visibility of small RNA derived from tRNA, and their functions in biological processes are being revealed. These include gene silencing, transcription and translation, epigenetics, and cell death. These properties have led to the implication of tsRNAs in various diseases. Although the current research mainly focuses on the role of tRNA-derived small RNA in cancer, there is mounting evidence that they are also strongly associated with cardiovascular disease, including cardiac hypertrophy, atrial fibrillation, heart failure, and myocarditis. Therefore, the regulatory role of tRNA-derived small RNA in cardiovascular disease will become an emerging therapeutic strategy. This review succinctly summarizes the characteristics, classification, and regulatory effect of tsRNA. By exploring the mechanism of tsRNA, it will provide a new tool for the diagnosis and prognosis of cardiovascular disease.

## Simple Summary

tsRNA (tRNA-derived small RNA), a class of non-coding RNA fragments generated through tRNA processing, plays significant roles in various diseases. This review systematically summarizes the classification, biogenesis, and functional characteristics of tsRNAs, with particular emphasis on their regulatory roles in programmed cell death. Furthermore, it explores the mechanistic involvement of tsRNAs in the pathogenesis of cancer, neurodegenerative diseases, and cardiovascular disorders. Notably, this article highlights the emerging potential of tsRNAs as diagnostic biomarkers and therapeutic targets for cardiovascular diseases. The elucidation of tsRNA-mediated regulatory networks provides novel approaches for the treatment of cardiovascular pathologies and lays a crucial foundation for advancing human health research.

## 1. Introduction

Transfer RNA (tRNA) is a small RNA that is highly abundant, accounting for 4–10% of cellular RNA. Most tRNAs have a relatively small number of nucleotides (nt) and possess a distinctive L-shaped tertiary shape. Such a tertiary structure is central to the process of protein synthesis, and its main role is reflected in the accurate transfer of specific amino acids to the ribosome [1]. Thus, the small RNA generated by specific cleavage also has a role in gene expression. This RNA is collectively called tRNA-derived small RNA (tsRNA).

**Figure 1:**
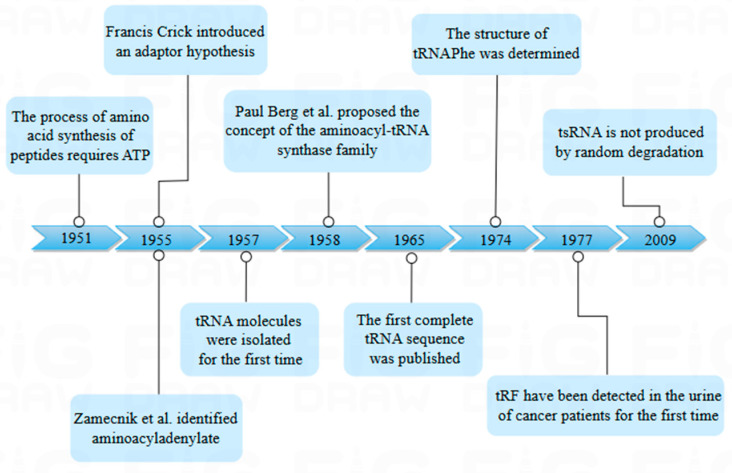
Historical timeline of transfer RNA (tRNA) research. The figure shows the time node when tRNA was first discovered and shows the research history of tsRNA.

tsRNA, micro RNA (miRNA), and small interfering RNA (siRNA) are all prevalent non-coding RNAs that play pivotal roles in the regulation of gene expression. However, their essential difference is the biogenesis process; tsRNA is cleaved by tRNA, while miRNA and siRNA are artificially synthesized or naturally produced for RNA interference [2]. tsRNA was first discovered in the urine of cancer patients in 1977 by Speer et al., using β-aminoisobutyric acid as a probe [3]. It was initially identified as a random degradation product of tRNA, which did not attract much attention. In 2009, Fu et al. identified the fragment produced by tRNA cleavage in the anticodon rings, which exist in many cells and tissues (Figure 1).

The fragment was found to be produced by the cleavage of different sites by an endonuclease called angiogenin (ANG) [4]. Due to the highly conserved sequence of the fragment and its ability to modify RNA and bind proteins, the current research has focused on the multifunctional role of tsRNA, which includes gene silencing, ribosome biogenesis, retrotransposition suppression, and epigenetic regulation [5]. The widespread and stable expression of tsRNA has been confirmed in a number of recent studies, and this expression is conserved among species. In addition, tsRNA is induced by cellular stress [6]. This indicates that tsRNA enables cells to respond quickly to stressful situations and participate in cell regulation, thereby maintaining cellular homeostasis and structural stability. In addition, tsRNA can regulate gene expression, consisting of cell proliferation, cell information transduction, and pyroptosis. Much evidence has illuminated that tsRNA regulates diseases by regulating cell death (CD) [2].

CD is crucial for maintaining cell homeostasis and fundamental biological functions, and it is essential in controlling abnormal CD for disease pathology. Since the first description of CD in the 1960s, CD has been distinguished by morphological changes and biochemical characteristics [7]. In general, the development of the disease is associated with apoptosis, necroptosis, ferroptosis, pyroptosis, and non-canonical death modalities. It is well known that the main feature of apoptotic cells is the change of mitochondrial osmotic pressure, the release of Cytochrome c (Cytc), and the generation of apoptotic bodies. Researchers believe that tsRNA can interact with Cytc, inflammatory proteins, etc., to regulate CD [8]. Research has demonstrated that tsRNA plays a critical role in programmed cell death and inflammatory responses, where the up-regulation of specific tsRNAs is associated with Caspase-1 activation, thereby promoting pyroptosis and the release of inflammatory factors [9]. The alteration in tsRNA expression may influence the activity of RIPK3, thereby modulating cellular responses to necroptotic signals [10]. Furthermore, tsRNAs may enhance or suppress the initiation and progression of cell death by modulating intracellular signaling pathways. To better control disease onset and prognosis, it is crucial to regulate the interactions between tsRNAs and key proteins involved in multiple cell death pathways.

With the continuous research on CD and non-coding RNA (ncRNA), such as tsRNA, it has been found that both of them may play a regulatory role in multiple pathways and play a key role in a variety of diseases. There is much evidence showing that the overexpression or knockdown of ncRNA plays a decisive role in CD. Furthermore, it intervenes in the development of a wide range of diseases. This includes cancer and cardiovascular disease (CVD), as well as inflammation. The regulation of tsRNA expression by drugs or gene intervention can be employed as a therapeutic approach for a series of diseases. Therefore, tsRNA has great potential to become a key target for disease treatment. In addition, ncRNA can also be used as biomarkers in CVD, cancer, and other diseases. This review presents a comprehensive analysis of the process by which tsRNA is formed and its involvement in regulating CD, and summarizes the different manifestations of tsRNA in a variety of diseases, especially the unique effect mechanism exercised in cancer and CVD.

## 2. Origin of Non-Coding RNA

Until the 1970s, the focus of genes was on the “active genes” of 2% of the human genome, while the remaining noncoding genes were considered “junk sequences”. However, as is evident from the Encyclopedia of DNA Elements (ENCODE) project, targeting only “effective genes” that encode proteins is far from enough. ENCODE reveals that tens of thousands of ncRNAs not only participate in the basic biological process that regulates growth and development but also significantly contribute to the control of gene expression. Moreover, due to the unique role of ncRNA, it is strongly associated with a variety of illnesses. Therefore, the exploration of ncRNA is indispensable to control the incidence and progression of illnesses [11].

In general, non-coding RNA can be classified into two major categories according to their shape characteristics: linear noncoding RNA and nonlinear non-coding RNA. Specifically, linear ncRNA is classified mainly on the basis of its length. NcRNAs consisting of 200 or more nt are called long non-coding RNA (lncRNA) while those with a length of less than 40 nt, consisting of miRNA, piwi-interactingRNA (piRNA), small interfering RNA (siRNA), short hairpin RNA (shRNA), and tsRNA, are called small RNA. The representative of nonlinear non-coding RNAs is a kind of circular non-coding RNA without the 5′ cap structure and the 3′ poly(A) tail structure [12]. Such a ring structure gives it higher stability [13]. The miRNA is an endogenous single-stranded small non-coding RNA (sncRNA) (Table 1). In the study of miRNA, it was discovered to be crucial to both normal bodily functions and disease processes by participating in biological processes such as epigenetics and transcriptional expression regulation [14]. For example, miR-1269 in gastric cancer (GC) acts on Ras association domain family member 9 (RASSF9) to regulate AKT-mediated pathways to promote the proliferation and migration of cancer cells, enter the GI-S cycle, and inhibit cell apoptosis [15]. The methyltransferase Enhancer of zeste homolog 2 (EZH2) can promote the methylation of miR-30d promoter H3K27me3, inhibit the transcription of miR-30d, and promote the progression of cardiac hypertrophy [16]. To date, more than 1880 unique human miRNAs have been identified, most of which target and inhibit the expression of hundreds of genes [17]. The reason why it can have such an obvious effect is likely the unique structure of precursor microRNA (pre-miRNA) after biogenesis. Pre-miRNA can bind to the Argonaute (AGO) protein to form the sRNA-induced silencing complex (RISC) [18]. The tsRNA we explored also has a similar biogenesis process to miRNA, with a unique silencing mechanism and the ability to bind RNA-binding protein (RBP).

## 3. The Origin of tsRNA

### 3.1. tRNA Biogenesis and Function

tRNA is the core of the translation process and the carrier of genetic information. Throughout the history of tRNA research, the connection of amino acids to their homologous tRNAs has puzzled researchers. It was not until 1951 that ATP was discovered to be required for the synthesis of peptides, and the first step in tRNA research was taken. It was in 1953 when Watson Crick first postulated the structure of DNA and then proposed the existence of a linker RNA molecule between DNA and proteins [19]. After that, the proposed adaptor hypothesis and the discovery of aminoacyladenylate promoted the study of tRNA. Hoagland et al. subsequently demonstrated the existence of this RNA and named it tRNA [20]. tRNA is a highly prevalent type of small RNA, making up from 4% to 10% of the total RNA population [21]. The length of tRNA ranges from 72 to 96 nucleotides, and its secondary structure adopts a "cloverleaf" conformation composed of the D-loop, TΨC-loop, anticodon loop, variable loop, and amino acid acceptor stem, terminating at the 3′ end with a single-stranded, highly conserved CCA sequence (typically at positions 74–76), which serves as the covalent attachment site for amino acids [22].

**Figure 2:**
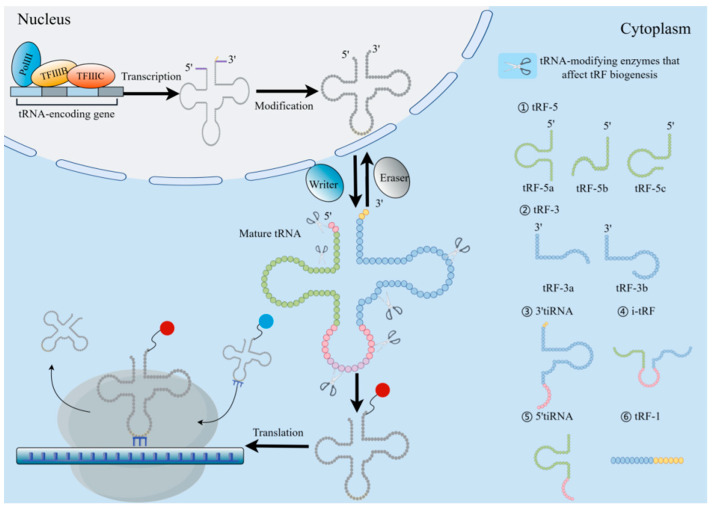
Origin and categorization of tRFs. TRF biogenesis begins with RNApol III and transcription factors TFIIIB and TFIIIC in the nucleus, and then the molecule is broken by RNase P and RNase Z at the 5′ and 3′ ends. The tRNA endonuclease was used to splice introns, attach the CCA sequence to the 3′ end, and further modify the intron to accomplish tRNA maturation. Under the action of the “writing protein”, the mature tRNA can participate in the translation process by aminoacylation. TsRNA is derived from tRNA through cleavage and serves as a regulator of gene expression at the transcription and translation levels. According to the cutting site of tRNA, tsRNA is mainly divided into tRF and tiRNA. The tRNA-cleaving endoribonucleases are depicted as scissors at their respective positions. The red/blue spheres carried by the 3′ tRNA termini are different amino acids.

tRNA biogenesis involves numerous processes, involving RNApol III-mediated transcription, ribonuclease modification CCA addition, and aminoacylation. First, the RNA polymerase transcribes the RNA as a precursor tRNA (pre-tRNA) with a 5′ leading sequence and 3′ tail sequence, then removes the 5′ end leading sequence by RNaseP and trims the 3′ end tail sequence by RNaseT and removes some intron sequences in the pre-tRNA. Finally, through the utilization of tRNA nucleotide transferase, the “CCA” sequence is inserted into the 3′ tail of the transferred RNA. At this time, the obtained sequence can be further post-transcriptional modification to form mature tRNA (Figure 2) [23].

Translation is the process of decoding the nucleotide sequence of an mRNA and generating a specific amino acid sequence. This process requires mature tRNA as a link to transfer amino acids to the ribosome. A total of twenty separate aminoacyl-tRNA synthetases (aaRS) are responsible for catalyzing the reaction, with each aaRS being specific for distinct amino acids [21]. The aaRSs provide raw materials for protein translation by catalyzing amino acids and corresponding tRNA to form aminoacyl-tRNA. aaRS catalyzes the covalent bonding of each amino acid to its corresponding tRNA in an aminoacylation process, where amino acids and ATP first bind to aaRS to form a starch adenylate intermediate, and aaRS binds to cognate tRNA to facilitate amino acid transfer to form acyl tRNA [24,25]. The acyl tRNA forms a complex with elongation factors (EF1A, EF-Tu) and subsequently transfers the amino acid to the A site of the ribosome and paired according to the codon–anticodon base pairing, ensuring accurate translation of the genetic information. However, there have been reports of gain-of-function (GOF) or loss-of-function (LOF) variants in aminoacyl-tRNA synthetase (aaRS)-encoding genes, which are associated with various genetic disorders and pathological processes. For instance, (I)LOF variants in mitochondrial AaRS genes are linked to hereditary diseases. Mitochondrial aaRSs play a critical role in mitochondrial translation, and genetic variants in these genes can lead to a range of inherited disorders. For example, mutations in genes encoding mitochondrial aaRSs can disrupt the synthesis of oxidative phosphorylation complex subunits, resulting in mitochondrial dysfunction-related diseases. (II)LOF variants in cytoplasmic aaRS genes are associated with neurological disorders. Mutations in the human cytoplasmic arginyl-tRNA synthetase (hArgRS) gene can lead to hypomyelinating leukoencephalopathy. Studies suggest that such mutations may disrupt the normal function of the enzyme, impairing myelination in neural cells and representing a typical LOF variant. Additionally, mutations in certain bifunctional aaRSs, such as KARS, have been linked to peripheral neuropathy, with potential mechanisms involving reduced enzymatic activity or loss of substrate-binding capacity [26]. Although cases directly reporting GOF variants in aaRS genes are relatively rare, studies suggest that the non-canonical functions of AaRSs, such as their involvement in inflammation and angiogenesis regulation, may be related to the multifunctionality of their structural domains. For instance, certain AaRSs, such as tyrosyl-tRNA synthetase, may exert pro-inflammatory or pro-oncogenic effects under specific conditions through domain-mediated interactions, implying potential GOF mechanisms [27].

The complex situation of tRNA modification is crucial for its biological function. The modification on the anticodon loop helps to adjust the interaction between the tRNA and the mRNA; the modification on the tRNA stem helps to maintain the structural stability of the tRNA. Current tRNA modifications are also not limited to maintaining tRNA function, and there is more evidence for its central position in tsRNA. Recent results illustrate that tRNA modifications have different effects in response to different cellular metabolic conditions and environmental factors. Abnormal modification of tRNA at any position may cause the occurrence of diseases containing CVD and cancer [28].

### 3.2. tRNA-Derived Fragments Biogenesis

Due to the extensive adoption of high-throughput RNA sequencing (RNA-Seq), tsRNA (also known as tDR or tRNA-derived fragment (tRF)) has been identified as an emerging class of functional sncRNA and has been connected to numerous clinical and physiological issues. tsRNA can be found in all domains of life, comprising archaea, bacteria, and eukaryotes [5]. These results can indicate that tsRNA has a rich and extensive function within and between cells. tsRNA is generated by specific endonucleases that cleave precursor or mature tRNA at different positions, depending on the tissue. The resulting tsRNA is typically 16–40 nt in length [29]. The length of tsRNA is similar to miRNA and piRNA and can be detected by RNA-Seq [30]. However, these small RNA fragments were initially thought to be random degradation products of tRNA [31]. Li discovered an instance of stress-induced tsRNA expression in 2008, which provided additional evidence that tsRNA is not subject to random degradation by tRNA. It was called sitRNA (stress-induced tRNA-derived RNA). Following that, Lee et al. reported the first functional tsRNA, designated tRF-1001, was reported and found to be strongly expressed in multiple cancers and, to a much lesser extent, in other tissues, associated with cell proliferation (Table 1) [32].

Studies have demonstrated that the nomenclature of tsRNAs typically follows the format X-tsRNA^AA-NNN^, where X denotes the tsRNA subtype (e.g., 1, 2, 3, 5); AA represents the three-letter abbreviation of the amino acid associated with the parental tRNA (e.g., Ala for alanine); NNN indicates the anticodon sequence of the corresponding tRNA (e.g., AGC). For instance, 1-tsRNA^Ala-AGC^ reflects a subtype 1 tsRNA derived from a tRNA-carrying alanine with the anticodon AGC [33]. Researchers have developed a variety of tsRNA expression databases. For instance, tReX is specifically designed for tsRNA analysis in Arabidopsis thaliana, PtRFdb contains over 5000 unique tsRNAs from ten plant species, GtRNAdb provides sequence information for human tRNAs and tsRNAs, and tsRBase includes more than 120,000 tsRNA sequences along with their expression profiles and functional information. These databases and tools offer crucial resources for investigating the biological functions and molecular mechanisms of tsRNAs, thereby advancing the research and applications of tsRNAs in various biological processes.

### 3.3. tRNA-Derived Fragments Classification

Structurally speaking, the abovementioned tRNA clover secondary structure appears as an L-shape in 3D. The L-shaped tertiary structure is tightly bound and stable but there are two relatively exposed restriction sites: the anti-codon at the L-end and the L-bending site. Therefore, the site of tRNA exposure may be the attack site of the stress signal, which can be broken and cleaved by enzyme-specific recognition to generate tsRNA [5].

Two main types can be distinguished from nuclear-encoded tsRNA based on their sequences: tRNA-derived stress-induced RNA (tiRNA or tRH) and tRF. When the RNaseA family member ANG cleaves mature tRNA in the anti-codon loop under stress circumstances, the resultant product is the tiRNA, also referred to as half of the stress-induced tRNA, with a length of 30–40 nt [4,34]. The tiRNA has two subclasses, which occur in opposite directions. Unlike 3′ tiRNA, 5′ tiRNA starts at the mature tRNA’s 5′ end and finishes in the anticodon loop [35].

Another class of derived fragments, tRF, are 14–30 nt in length. Specifically, they are found at the termini of mature or main tRNA transcripts. Since the nt fragment length of tRF is similar to that of miRNA, it has attracted much attention [36]. According to its position in relation to the structure of tRNA, tRF is classified in a specific manner and thus can be divided into four different categories, with both ranges overlapping with mature tRNA and tRF overlapping with precursor tRNA, including (I) 5′tRF or tRF-5, (II) 3′-tRF or tRF-3, and (III) i-tRF or internal tRF. i-tRF is entirely encompassed within the spectrum of fully developed tRNA and was initially identified through the examination of next-generation sequencing (NGS) data [37]; it is the most numerous subcategory in tRF [38]. (Ⅳ) tRF-1 is a tRF including a constituent 3′ tail sequence (Figure 2) [34]. Among them, the subclasses of tRF are produced by Dicer, endonuclease Z(RNaseZ/ELAC2), and other enzymes. As opposed to tRF-1, which is produced from the 3′ end of the primary tRNA transcript, tRF-5 and tRF-3 are produced by the mature 5′ and 3′ ends of tRNA, respectively [32]. The D-loop of tRNA or the stem area between the D-loop and the anticodon loop can be cleaved to produce tRF-5, which is a fragment that is between 1 and 30 nucleotides in length. When the length of the subclass is taken into consideration, the tRF-5 is further subdivided into the tRF-5a (14–16 nt), tRF-5b (22–24 nt), and tRF-5c (28–30 nt) categories [39]. The length of tRF-3 is approximately 18 or 22 nt. Therefore, it has two subclasses (tRF-3a or tRF-3b), and the cleavage sites of both tRF-3 are located in the TCC loop [40]. The tRF-1 is a small fragment containing the pre-tRNA 3′ tail sequence.

### 3.4. Regulation of tsRNA Biogenesis

Modifications to tRNA, which can be used to influence the fate of tsRNA, are responsible for regulating the synthesis of tsRNA. The tRNA modification helps to improve structural stability; for example, endowing the base or ribose structure with rigidity, creating either a water-repelling or water-attracting setting for the nearby structure [41]. In addition, through specific chemical modifications, it also has the effect of promoting tsRNA biogenesis [42]. Some studies have demonstrated that the methylation of tRNA can protect tRNA from ANG cleavage and down-regulate tsRNA content. Among several tRNAs (e.g., tRNAAsp, tRNAVal, tRNAGly, and tRNALeu), the addition of 5-methylcytosine (m5C) modification dependent on the methyltransferases DNA methyltransferase 2 (DNMT2) and NOP2/Sun RNA methyltransferase 2 (NSUN2) increased tRNA stability in Drosophila and mouse. The loss of either DNMT2 or NSUN2 eliminates m5C, making it more susceptible to cleavage to generate tsRNA [43]. Queuosine (Q) modification of the QTRT1 gene acts at the swing anticodon position of tRNA, and the Q modification improves the stability of tRNA [44]. There have been recent reports suggesting a synergistic effect of Q modification. C38 Q-modified tRNA can promote the m5C methylation modification of DNMT2, which is more conducive to reducing the production of tsRNA [45]. Furthermore, the 2′-O-methylation of C34 in humans limits the synthesis of tsRNA and prevents the cleavage of tRNA carried out by stress-induced ANG (Table 1) [46].

In addition to inhibiting tsRNA biogenesis, some RNA modifications can also facilitate tsRNA generation. A simple example is that when the production of methyltransferase is decreased, it directly results in the demethylation of tRNA, which makes tRNA more sensitive to ANG. This was confirmed in neuroblastoma cells, where AlkB homolog 3 (ALKBH3) acts as a demethylase and its overexpression removes N1-methyladenosine (m1A) and 3-methylcytidine, promoting tRNA cleavage by ANG and tiRNA production [47]. To prevent the decrease of tsRNA content, pseudouridine (Ψ) is another common modifier of tRF regulation. The deletion of stem cell-specific pseudouridine synthetase (PUS7) down-regulated the amount of 5′tRF produced by tRNAAla, tRNACys, and tRNAVal with 5′ terminal oligopurines (TOG) [48]. In addition, 7-denitrogenous guanosine on tRNAHis, tRNAAsn, tRNATyr, and tRNAAsp anticodons can protect tRNA from the cracking of ANG, thus maintaining the biogenesis of the corresponding tsRNA. There are also cases of regulation of biogenesis by modification in bacteria. Two kinds of tsRNA are generated when bacterial tRNA is cut in half by a nuclear toxin containing the Q anticodon. However, the generated tsRNA can be re-repaired into a tRNA/Hen1 heterotetramer by polynucleotide kinase phosphatase (PNKP), and 2′-O methylation is modified at the cleavage site to strengthen the stability of tRNA from repeated cleavage. The tRNA modifications are not immutable to tsRNA biogenesis, and the rules are not fully understood. In summary, it has been demonstrated that the epigenetic modification of tRNA regulates tsRNA biogenesis [49].

### 3.5. Comparison of tsRNA and miRNA Biogenesis

The biogenesis of miRNA initiates with the transcription of the miRNA gene within the nucleus, which is called the new transcript of primary miRNA (pri-miRNA). After pri-miRNA is processed by RNase III enzymes in the nucleus, 60–70 nt pre-miRNA are transferred to decrease to form a double chain, and then they can be transferred to the AGO protein, which will then be responsible for forming an effective RISC. And between different types of AGO proteins, miRNA exhibits a distinct affinity for AGO2, as evidenced by its structural characteristics [50]. tRF-5s and -3s, despite not being processed by Dicer, exhibit a close association with the AGO protein family, particularly AGO1, 3, and 4. AGO Photoactivatable-ribonucleoside-enhanced crosslinking and immunoprecipitation (PAR-CLIP) and the cross linking-ligation and sequencing of hybrids (CLASH) data demonstrate that tRF-3 (and to some extent, tRF-5) recruits target RNAs to AGO protein complexes through complementary sequences. This recruitment leads to the modulation of target expression or function, similar to the action of miRNA (Table 1) [40]. Although there are currently no clear reports on the half-life of tsRNA, based on the characteristics of its biogenesis, we speculate that the half-life of tsRNA is similar to that of miRNA.

In addition, the experiment results illustrated the chimeric RNA between miRNA and its target RNA, which is produced by connecting two RNAs, and they are interrelated in the AGO1 protein. Many chimeras have been regarded as tRF fragments with 3′ non-translational regions (UTR) connected to annotated protein-coding genes. It is noteworthy that there are two to three times as many tRF-3 target RNA chimeras in AGO1 sediment as miRNA target RNA chimeras, which is remarkable. All in all, the experimental data clearly show that tRF is related to target RNA, and tsRNA is similar to miRNA biogenesis [39].

However, studies have shown that tsRNAs and miRNAs can compete with each other by co-targeting specific mRNAs, thereby influencing gene expression levels. The recognition of their shared targets relies not only on sequence complementarity but is also modulated by various factors, including the intracellular environment and transcriptional regulation [51]. Furthermore, the expression levels of tsRNAs and miRNAs may vary under different physiological and pathological conditions, leading to competitive interactions in the regulation of shared targets. This competition can ultimately influence cellular functions and fate [52]. For instance, Tan et al. discovered that the L-NAME-induced paternal hypertension model alters the sperm tsRNA profile. Specifically, the aberrantly expressed tsRNA-00951 and tsRNA-00051 may function as competitive endogenous RNAs (ceRNAs), competing with miR-128-1-5p to regulate the expression of the target gene Agap1 [53]. The constructed tsRNA–miRNA–mRNA regulatory network provides a novel direction for exploring therapeutic strategies for various diseases.

### 3.6. The Action Mechanism of tsRNA

tsRNA is a small RNA that widely exists in viruses, archaea, bacteria, protozoa, plants, mice, and humans and runs through the entire evolutionary tree. Although tRF research is still in its infancy, there is emerging evidence for multiple functional roles of tsRNA in a subset of organisms. Next, its functions will be elaborated from the aspects of gene silencing and transcription regulation.

**Figure 3:**
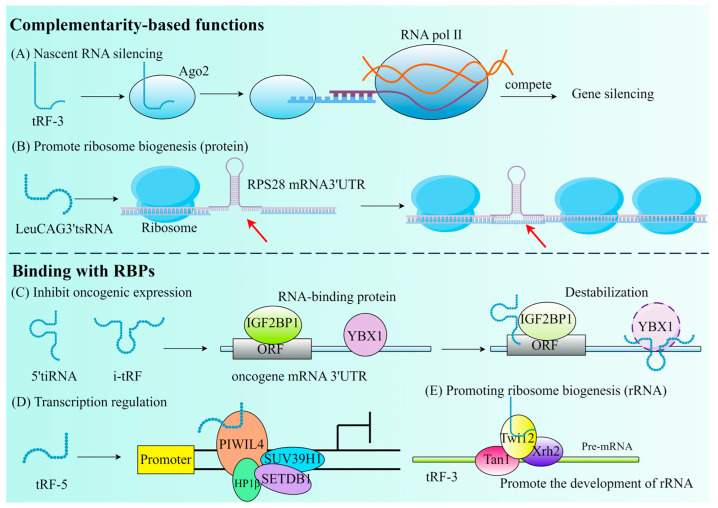
The primary roles of tRFs that are determined by their complementarity or their binding with RBPs. (**A**) Nascent RNA silencing. (**B**) Promote ribosome biogenesis. The red arrow emphasizes that tsRNA binds to the mRNA 3′UTR. (**C**) Inhibit oncogenic expression. (**D**) Transcription regulation. (**E**) Promoting ribosome biogenesis (rRNA).

As mentioned earlier, tRF has an affinity with the AGO protein family. According to findings from recent research, a new method for RNA silencing (NRS) is the Dicer-dependent tRF protocol, which is different from post-transcribed gene silencing (PTGS) and transcriptional gene silencing (TGS). The silence of Dicer-dependent tRF begins in the nucleus and has no effect on the transcription of the target gene. The distinguishing characteristic of tsRNA is its ability to attach to the AGO2 protein and identify RNA targets by utilizing base pairing similar to miRNA and to directly cut the new RNA in a sequence-specific way, resulting in the downward adjustment of the target gene and preventing its translation into proteins (Figure 3A) [54].

Due to the conservative evolution of tsRNA, NRS may be a universal mechanism for gene expression regulation in eukaryotes. If it can silence proto-oncogenes, inflammatory cytokines, and other pathogenic genes, NRS can become a new method for disease treatment. The study by Green et al. discovered that IL-1β stimulates chondrocytes to produce tRF3003a, which suppresses JAK3 gene expression by attaching to the AGO2–GW182 protein complex. This, in turn, reduces JAK, which reduces the expression of IL-6, hence reducing the progression of osteoarthritis [55]. The initiation phase of translation in eukaryotic cells involves a multitude of translation initiation factors (eIFs), which play an essential role in the translation process. 5′tiRNAAla and 5′tiRNACys contain a TOG motif and a G-quadruplex structure (RG4) at their termini, which can facilitate the release of eIF4 from the m7G cap of mRNA, thereby inhibiting translation. Additionally, 5′tiRNAs can promote the phosphorylation of eIF2, which facilitates the formation of stress granules (SGs) and leads to a delay in translation [56].

In addition, tRF can also participate in post-transcriptional gene expression by interacting with RBP, separating RBP from the target RNA, thereby affecting RNA stability. It has been demonstrated that by substituting the 3′ untranslated regions of RBPs, a set of i-tRFs generated from tRNAGlu, tRNAAsp, tRNAGly, and tRNATyr can specifically modify Y box binding protein 1 (YBX1) through post-transcriptional mechanisms, thereby fulfilling the opposite function. Instead of its stabilizing role in breast cancer (BC), it plays an inhibitory role in the transcription of proto-oncogenes, thereby lowering oncogene activity and eventually preventing the growth of cancer (Figure 3C) [57]. It is not uncommon for tRF to bind to RBP to inhibit transcription. tsGlnCTG can combine with IGF2BP1 (an RBP of c-Myc mRNA) in mouse embryonic stem cells to promote its differentiation [58]. However, the selection of tsRNAs to interact with AGO proteins or RBPs is influenced by multiple factors: (1) The type and origin of tsRNAs: tRF-3 and tRF-5 are more likely to bind to AGO proteins, while tiRNA and tRF-1 tend to interact with RBPs. (2) Cellular environment and stress conditions: under stress conditions, tsRNAs are more likely to interact with RBPs to regulate translation or stress granule formation, whereas in RNA silencing pathways, tsRNAs preferentially bind to AGO proteins. (3) Characteristics of target mRNAs: if target mRNAs require sequence-specific cleavage for silencing, tsRNAs will bind to AGO proteins; if target mRNAs function through stability or translational regulation, tsRNAs tend to interact with RBPs [59].

tRF can be found either through competitive inhibition of translation initiation complexes (Figure 3D) or through aberrant modification during ribosome biogenesis (Figure 3B,E). tRF is essential for the CD process. In mouse embryonic fibroblasts (MEF), the regulation of the mitochondrial permeability transition pore can release large amounts of Cytc to induce apoptosis. Saikia et al. thus proposed that the induction of 5′ and 3′-tiRNA production sequesters Cytc in ribonucleoproteins, thereby protecting MEF from the threat of apoptosis [8]. Therefore, it is possible to hypothesize that tRF also serves the purpose of controlling CD at the same time, which provides a new treatment strategy for tumors and CVD.

## 4. Role of tsRNA in Regulating Cell Death

**Figure 4:**
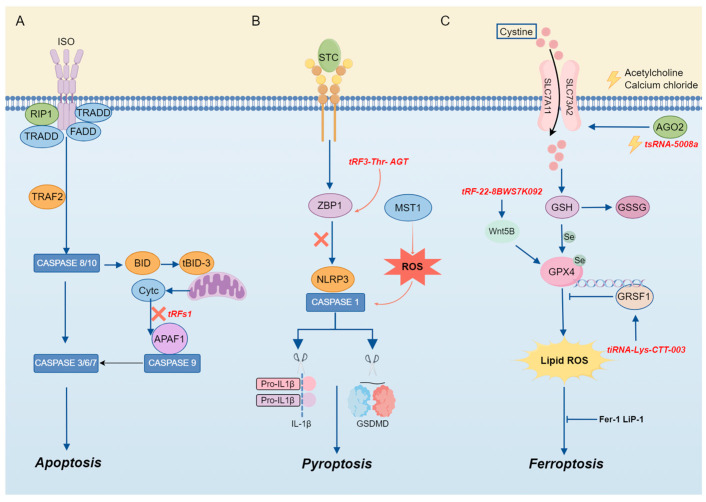
The mechanism of tsRNA in cell death. (**A**) TsRNA is involved in apoptosis. (**B**) TsRNA is involved in pyroptosis. (**C**) TsRNA is involved in ferroptosis.

In this context, we mainly discussed the regulatory functions of tsRNA in apoptosis, pyroptosis, necrosis, ferroptosis, and autophagy, which will provide new avenues for the treatment of tumors, CVDs, and other conditions (Figure 4).

### 4.1. tsRNA and Apoptosis

**Figure 5:**
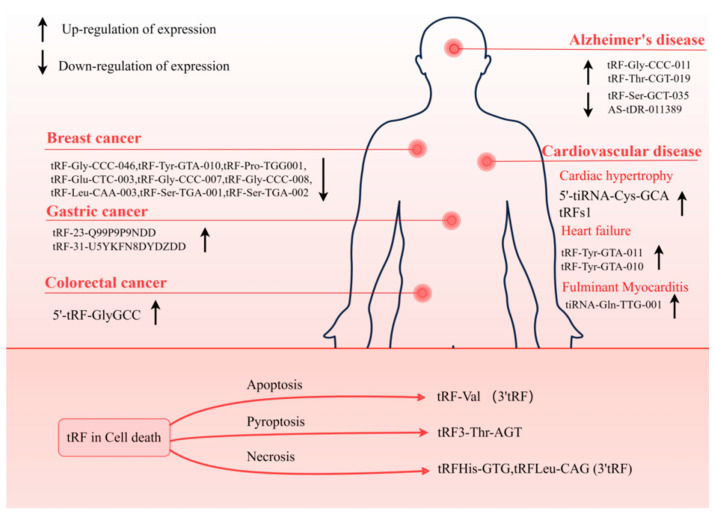
tRFs involved in diseases. tRF can be used as a biomarker for a variety of diseases, consisting of neurodegenerative diseases, cancer, and CVD. The tRF plays a regulatory role in CD. Detection of these tRFs is beneficial to the diagnosis and prognosis of the disease.

Here, we summarized that tsRNA can regulate CD and contribute to the onset and advancement of diseases or conditions (Figure 5).

tiRNA is an essential component in the cellular apoptotic pathway. Apoptosis occurs if cells are damaged beyond the scope of self-repair. During apoptosis, mitochondria undergo biochemical changes that result in the release of Cytc from the mitochondrial membrane space [60]. Previous studies have shown that apoptosis induced by hyperosmotic stress can be reversed by Ang-induced tiRNA, as mentioned above. The mechanism can be attributed to the cleavage of tRNA by ANG under stress, which leads to the accumulation of tiRNA and then interacts with Cytc released from mitochondria during apoptosis to competitively inhibit the apoptotic protease-activating factor 1 (APAF1), thereby inhibiting the formation and activity of apoptotic bodies to realize the regulation of apoptosis [8] (Figure 4). Previous evidence has illustrated that there is a clear correlation between cardiac hypertrophy and tRF, and tRFs are abundant in the semen of mice with cardiac hypertrophy [61]. Therefore, when researchers injected the tRF of high-fat mouse sperm into normal fertilized eggs, they found that in MEF, elevated tsRNA competitively binds to Cytc and prevents it from binding to APAF1. The activation of caspase-9 and caspase-3 is the mechanism by which this procedure prevents the induction of apoptosis [62].

### 4.2. tsRNA and Pyroptosis

Pyroptosis is a programmed CD mediated by caspase-1, a key enzyme that coordinates biological effects [9]. It exerts a regulatory influence on the development and progression of inflammation or illness. According to the findings of Sun et al., acute pancreatitis (AP) is characterized by the aberrant expression of tRF3-Thr-AGT. Through the rescue of pyroptosis and the maintenance of cell homeostasis, AP can be alleviated through the overexpression of tRF3-Thr-AGT. The mechanism by which it exerts its effects involves tRF3-Thr-AGT targeting the 3′ UTR of the Z-DNA-binding protein, a key regulator of inflammasome activation, leading to the degradation of ZBP1, which can activate the NLRP3 inflammasome, thereby alleviating AP and controlling disease progression. These results suggest that tRF3-Thr-AGT has a defensive function in the progression of AP by controlling pyroptosis and inhibiting inflammation [63] (Figure 4).

Furthermore, research has demonstrated that the primary monomer tectorigenin (TEC) in blueberries can activate autophagy, significantly reversing the release of pyroptosis and inflammatory substances induced by non-alcoholic steatohepatitis (NASH). The researchers sequenced the small RNAs of hepatocytes treated with TEC and identified 122 differentially expressed tsRNAs. These differentially expressed tsRNAs are primarily involved in the “PI3K-Akt signaling pathway”, “Wnt signaling pathway”, “MAPK signaling pathway”, and “Foxo signaling pathway”. Among them, tRF-47-58ZZJQJYSWRYVMMV5BO exhibited significant differential expression. Ultimately, it was demonstrated that TEC enhances autophagy and attenuates pyroptosis by activating the expression of tRF-47-58ZZJQJYSWRYVMMV5BO [64].

### 4.3. tsRNA and Necrosis

It is well known that the excessive release of glutamate can cause necrosis of neurons. In past studies, the strategy for glutamate receptors did not achieve ideal clinical results. Therefore, looking for downstream mechanisms for glutamate receptor activation may provide a better strategy to inhibit necrosis. It has been proven that inhibiting H3K4 methylation will prevent the necrosis of neurons. During neuronecrosis, tRF is enriched and shows strong cytotoxicity. In this case, the synthesis of nascent proteins is inhibited. Subsequently, tRFHis-GTG and tRFLeu-CAG (tRF-3) were found to be cleaved by AGO2 after interacting with endogenous AGO2 protein. However, if AGO1/2 RNAi is interfered with, neuronal necrosis can be rescued. Although this effect is not necessarily produced by tRF, it may provide a new strategy for neuronal necrosis [65].

### 4.4. tsRNA and Ferroptosis

Ferroptosis is a form of iron-dependent programmed cell death, characterized by excessive lipid peroxidation and the disruption of cellular metabolic and intracellular redox homeostasis [66]. The regulation of ferroptosis involves multiple molecules and signaling pathways, encompassing negative regulators such as glutathione peroxidase 4 (GPX4), heat shock protein β-1 (HSPB1), and nuclear factor erythroid 2-related factor 2 (NRF2), as well as positive regulators such as NADPH oxidase and p53. These regulatory factors modulate ferroptosis by either restricting the generation of reactive oxygen species (ROS) and reducing cellular iron uptake or by promoting ROS production and inhibiting the expression of SLC7A11 [67] (Figure 4).

Emerging evidence highlights that tsRNAs play a significant role in the regulation of ferroptosis. The expression of tsRNA-5008a in HL-1 cells is elevated following induction by angiotensin II (Ang II). tsRNA-5008a modulates SLC7A11, a marker associated with ferroptosis, through AGO2 targeting, thereby increasing fibrosis and ferroptosis in myocardial tissue, which in turn exacerbates the progression of atrial fibrillation (AF) [68].

In addition, numerous tsRNAs are involved in the regulation of ferroptosis. For instance, tRF-22-8BWS7K092 derived from alveolar macrophage exosomes targets Wnt5B to activate the Hippo signaling pathway, leading to ferroptosis in alveolar epithelial cells and contributing to the pathogenesis of acute lung injury [69].

In gastric cancer (GC), tRF-23-Q99P9P9NDD is up-regulated and targets the 3′ untranslated region (UTR) of short/branched-chain acyl-CoA dehydrogenase (ACADSB), thereby suppressing ACADSB expression. This downregulation of ACADSB inhibits fatty acid metabolism, promotes lipid accumulation, and accelerates the progression of ferroptosis, thereby contributing to the development of GC [70].

In cisplatin-induced acute kidney injury, it has been confirmed that tiRNA-Lys-CTT-003 is a non-coding RNA (ncRNA) with a protective role. GRSF1, a RNA-binding protein, can bind to the AGGGA sequence in the 5′ untranslated region (UTR) of GPX4 mRNA, thereby facilitating the transfer of mRNA to activated polyribosomes, enhancing the translation of GPX4, and thereby mitigating the development of ferroptosis [71]. Research has demonstrated that tiRNA-Lys-CTT-003 exhibits a high affinity for GRSF1. The interaction between these two molecules facilitates the expression of GPX4, thereby alleviating renal injury-induced ferroptosis [72]. Additionally, tsRNA-13502 can mitigate the damage induced by epigallocatechin gallate (EGCG) in non-small cell lung cancer (NSCLC) cells by counteracting ferroptosis [73].

Supported by numerous cases, there is compelling evidence to suggest that tsRNAs hold promise as therapeutic targets for mitigating ferroptosis. Future research should delve deeper into the redox-related regulatory networks of tsRNAs to elucidate their complex regulatory mechanisms in ferroptosis and their roles in both health and disease.

### 4.5. tsRNA and Autophagy

Autophagy is the primary dynamic recycling system within cells, capable of transporting cytoplasmic materials to lysosomes for degradation [74]. In gliomas, tRNA-Cys-GCA-derived tsRNAs (e.g., tRFdb-3003a and tRFdb-3003b) are markedly down-regulated. These tsRNAs regulate the expression of the proto-oncogene VAV2 by binding to its 3′UTR, thereby affecting the autophagy pathway [75].

Generally, autophagy plays a cytoprotective role in cellular homeostasis. However, an increasing body of evidence suggests that excessive autophagy can promote the progression of diseases. In traumatic brain injury (TBI), the inhibition of autophagy can minimize the damage caused by TBI. Research has shown that the expression of the ubiquitin ligase Rnf6 is up-regulated in TBI. Yang et al. found that tRF-64-85-Leu-AAG-M4 can directly interact with the target Rnf6 to inhibit its expression level, thereby controlling autophagy and alleviating the damage caused by TBI [76].

However, in the process of tsRNA playing a role, the researchers found that tsRNA can be packaged into extracellular vesicles (EVs) and delivered through the microenvironment in vivo to play a regulatory role. EVs derived from M5006 macrophages carrying tsRNA-5006c possess the capability to alleviate the osteogenic differentiation of aortic valve interstitial cells (AVICs), which plays a crucial role in the progression of calcific aortic valve disease. Researchers have found that tsRNA-5006c likely targets Wnt2 and Btrc to participate in the Hippo signaling pathway, Ntrk1 and Mknk2 to participate in the MAPK signaling pathway, Ppp5r2e and Camk3b to participate in the Wnt signaling pathway, and Akt9 and Mapk4 to participate in the Ras signaling pathway, thereby modulating autophagy [77]. Notably, bone marrow mesenchymal stem cells (BMSCs) and their extracellular vesicles (EVs) demonstrate significant therapeutic effects across different stages of chronic obstructive pulmonary disease (COPD), with tsRNAs playing a pivotal regulatory role in this process [78]. Furthermore, seminal plasma EV-derived tRF-Val-AAC-010 and tRF-Pro-AGG-003 [79] may serve as potential non-invasive biomarkers for predicting successful sperm retrieval in patients with non-obstructive azoospermia (NOA). These findings collectively underscore that tsRNAs possess distinct transport mechanisms to modulate disease-related signaling pathways, thereby regulating disease progression or pathogenesis (Table 2).

## 5. The Role of tsRNA in Cancer

Recently, ncRNA has come to prominence in altering cellular processes. Different classes of ncRNA have different roles in cancer progression. In 2018, Pliatsika et al. launched MINTbase v2.0 by examining the entire dataset of the Cancer Genome Atlas (TCGA) up until October 2017. This dataset consisted of 26,531 distinct collections of human tRFs [38]. The tRF exhibits several biological functions throughout the onset and progression of cancer. Therefore, we will summarize the biological roles of tRF in different cancers and the related mechanisms.

### 5.1. Early Diagnosis

Several investigations have shown evidence of the participation of tRF in the development and advancement of tumors, either by its distinct mechanism of gene silencing or its capacity to attach to RBP. In addition, tRF is stable and abundant within biofluids including serum and urine, so much data have shown that when diagnosing malignant tumors, tRF might be utilized as a marker. Zhang et al. discovered that patients with early breast cancer had lower levels of tRF-Gly-CCC-046, tRF-Tyr-GTA-010, and tRF-Pro-TGG001 in their serum and tissues. Moreover, tRF was found to enhance the detection of carcinoembryonic antigen (CEA), carbohydrate antigen 125 (CA125), and carbohydrate antigen 153 (CA153), three traditional breast cancer biomarkers [80]. In addition, the plasma of early-stage BC patients showed a considerable down-regulation of six 5′-tRFs. This finding further supports the clinical relevance of tRFs as biomarkers [81]. Similar examples have been reported in other cancers, such as the increased expression levels of 5′-tRF-GlyGCC in the serum of colorectal cancer (CRC) patients [82], and significantly overexpressed tRF-23-Q99P9P9NDD [83] and tRF-31-U5YKFN8DYDZDD [84] (Figure 5) are not only related to the stage of tumor development and vascular invasion but also directly connected to the capacity of tumor cells to invade and migrate. Such cases can establish the important role of tRF as a cancer biomarker.

### 5.2. Means of Treatment

The role of tRF is not only limited to early diagnosis but also more and more evidence shows that it is involved in the proliferation of tumor cells, suppressing the activity of genes that inhibit the growth of tumors and other biological processes. It will become the target of cancer treatment, providing a novel avenue for cancer treatment. At the mechanistic level, tRF mainly plays a role in two ways: miRNA-like base pairing to find RNA targets and down-regulate the expression of target genes and binding with RBP to regulate signaling pathways. Mo et al. discovered that 5′-tiRNAVal and tRF-17-79MP9PP act as tumor suppressors in breast cancer (BC). By interacting with AGO2, they induce the assembly of the RNA-induced silencing complex (RISC), suppress the activation of tumor-associated signaling pathways, and thereby inhibit the proliferation and metastasis of BC cells [85,86]. In GC, there is also some tRF-mediated post-transcriptional gene silencing that is AGO-dependent. For example, by directly attaching to the 3′UTR of FBXO47, tRF-3019a can engage with AGO2 to suppress the expression of FBXO47 and enhance the invasion ability of tumor cells [87]. The tRF-3017A and tRF-24-V29K9UV3IU bound to AGO2 in the same way, and then specifically bound to the 3′UTR of NELL2 and GPR78mRNA [88,89]. In addition, tRF mimetics derived from the 5′ -terminus of tRNAHis (GUG) of taxus chinensis also showed potent anticancer activity by directly targeting oncogenes, even comparable to that of paclitaxel [90].

However, there are also many examples of tRF that exert their effector mechanisms by acting as protein bait to interact with RBP. YBX1 (RNA-binding proteins called RNA translation and stability regulators) could be competitively bound by a set of i-tRFs from tRNAGlu, tRNAAsp, tRNAGly, and tRNATyr, according to research by Goodarzi et al. with endogenous transcripts in BC patients to eliminate the stabilizing effect of YBX1. This results in the reduction of mRNA stability and the down-regulation of oncogene expression, which ultimately inhibits the metastasis and spread of cancer cells [57]. Another homolog of i-tRF tRFGlu, tRF3E, also acts as a breast cancer repressor by interacting with nucleolin, trapping transcripts from p53 mRNA and blocking the translation process [91]. In addition, Yang et al. discovered that in non-small-cell lung cancer (NSCLC) cells, AS-tDR-007333 interacts directly and very selectively with heat shock proteins (HSPB1). Therefore, we can enhance the histone modification in the promoter of the target gene MED29 after HSPB1 activation to stimulate the expression of the target gene. It can also participate in the regulation of transcriptional expression and promote the development of tumor cells by stimulating the expression of the transcription factor ELK4 of the target gene [92]. The research on tRF in cancer is still ongoing, and the current results have demonstrated a significant association between tRF and cancer. In the process of research, researchers continue to search for bioactive tRF. Analyzing the molecular mechanism of tRF in different cancers and how it exerts its effect also provides targets for the cure of cancer, which has aroused the potential of tRF as an effective therapeutic molecule.

The tRF has emerged as a functional molecule that can aid in cancer therapy because of its similarity to miRNA. As mentioned above, tRF has a silencing mechanism similar to miRNA. Therefore, tRFs may serve as potential therapeutic candidates, including interference-based therapies, anti-miRNA oligonucleotides (AMOs), locked nucleic acid (LNA)-modified oligonucleotides, and miRNA antagonists (e.g., antagomirs) [23]. King et al. employed LNA to hinder the growth of cancer-causing tsRNA, specifically targeting 3′tRF-LeuCAG. This intervention effectively triggered programmed cell death (apoptosis) in a mouse model of hepatocellular carcinoma [93]. For tRF with a tumor inhibitory effect, tRFs may serve as potential therapeutic agents. Notably, endogenous tRFs often require chemical stabilization (e.g., 2′-O-methylation), whereas synthetic tRF analogs can be pre-modified to enhance pharmacokinetics and therapeutic efficacy [23].

## 6. The Role of tsRNA in Neurodegenerative Diseases

Neurodegenerative diseases are characterized by neuronal damage and the structural and functional collapse of neural networks. However, under the pathological conditions of various neurodegenerative diseases, we have identified that the abnormal expression of proteins such as Tau, amyloid-β, α-synuclein, huntingtin, and TDP-43 is a primary driver of neurodegenerative disease onset [94]. Numerous studies have demonstrated that tsRNAs play a significant role in the pathogenesis and progression of neurodegenerative diseases. Parkinson’s disease, a common chronic progressive movement disorder, is primarily characterized by symptoms such as bradykinesia, tremors, and postural instability. Magee et al. [95] identified distinct tsRNA expression profiles in cerebrospinal fluid (CSF), the prefrontal cortex, and serum from PD patients compared to healthy controls. Zhang et al. [96] compared the senescence-accelerated mouse-prone 8 (SAMP8) model with the senescence-accelerated mouse-resistant 1 (SAMR1) model and found eight differentially expressed tRFs associated with synaptic formation and synaptic vesicle cycle pathways. Additionally, they reported that AS-tDR-0111389 interacts with the endogenous CaMKII inhibitor Camk2n1, thereby affecting synaptic CaMKII-NMDAR binding and LTP regulation, while AS-tDR62 targets Rpsa to promote the production and internalization of neurotoxic amyloid-β peptides [96]. However, in amyotrophic lateral sclerosis (ALS), which is characterized by muscle and limb paralysis, 5′tiRNAAla and 5′tiRNACys can form G-quadruplex structures to protect neurons in a YB-1 protein-dependent manner [97]. Additionally, ANG enhances neuronal resistance to stress, hypoxia, and cell death induced by the withdrawal of trophic factors [98]. These findings suggest that tsRNAs hold significant potential as biomarkers for neurodegenerative diseases, while also representing promising therapeutic targets for these conditions.

## 7. The Role of tsRNA in Cardiovascular Disease

CVD is the leading cause of death globally, with an increasing incidence as a result of aging populations and lifestyle factors. Among the most common cardiovascular diseases are heart muscle damage (e.g., myocardial ischemia, MI), atrial fibrillation (AF), heart failure (HF), and Coronary Heart Disease (CHD). According to the American Heart Association (AHA) [99], approximately 1.4 million deaths occurred from cardiovascular diseases worldwide in 2021, and the prevalence of heart disease among younger individuals has increased over time. This underscores the urgent need for early detection and appropriate treatment of cardiovascular diseases.

### 7.1. Cardiac Hypertrophy

ncRNA has important and diverse molecular functions in cells, so they can act as important regulators under both physiological and pathological conditions. tRF exerts various critical functions in cells by generating different isoforms through different cleavage modes. Early studies identified 5′tRNA halves derived from Val- and Gly-tRNA cleavage as abundant RNA species in murine cardiac tissue [4,100]. Specific tRFs were identified in human heart tissue as well (Figure 5). It was shown that human heart tissue had tRF-3, which was derived from Arg- and Gln-tRNA, as well as tRF-5, which was derived from Gly- and Cys-tRNA [101]. In addition, 5′-tiRNA-Cys-GCA was found to inhibit the abnormal development of vascular smooth muscle cells (VSMCs) by directly binding to the signal transducer and activator of transcription 4 (STAT4) to down-regulate its expression [102]. tRFGlnCTG is a crucial regulator in promoting VSMC proliferation [103]. Excessive proliferation of VSMC leads to myocardial hypertrophy and even heart failure (HF).

These results establish a foundation for the correlation between tRF aberrant expression and the malfunction of the cardiovascular system, making tRF research in CVDs an emerging hotspot. For example, in the study of Shen et al. [61], tRF was found to respond to the stress of myocardial hypertrophy. During the course of the study, tRF was found to be highly enriched when exposed to the stress process of myocardial hypertrophy. In contrast, tRFs1 derived from tRNA-Gly-CCC showed the highest expression in response to stress. It has been demonstrated that tRFs1 binds to the 3′UTR of the hypertrophic regulator Timp3 mRNA, which in turn inhibits the production of Timp3 and, ultimately, results in cardiomyocyte hypertrophy. More importantly, this study suggests that the expression pattern of tRF is closely related to intergenerational inheritance. tRFs play a crucial role in intergenerational inheritance, not only participating in the regulation of gene expression but also potentially influencing the phenotypic traits of offspring through epigenetic mechanisms. Studies have shown that parental environmental factors can alter the epigenetic state of offspring by affecting the expression of tRFs. For instance, metabolic disorders induced by a high-fat diet can be transmitted to offspring through modifications and expression changes of tRFs in sperm [104]. After being modified in the parental germ cells, tRFs can be transmitted to offspring during fertilization, influencing embryonic development and subsequent physiological traits [105]. This underscores that tRFs serve as a critical bridge linking environmental and genetic information, highlighting their significant role in hereditary susceptibility to diseases.

Previous studies have identified tsRNA expression profiles in patients with cardiac hypertrophy and validated real-time tRF expression in Ang II-stimulated H9c2 cells using RNA sequencing. tRF-21-NB8PLML3E was found to ameliorate Ang II-induced hypertrophy and thus could be considered as a biomarker. In order to further elucidate the molecular mechanism of tRF-21-NB8PLML3E, the target genes of tRF-21-NB8plML3E were shown to be strongly associated with ribosome biogenesis, as indicated by the results of Gene Ontology (GO) analysis and the Kyoto Encyclopedia of Genes and Genomes (KEGG) pathway analysis. These findings indicate that tRF-21-NB8PLML3E has the ability to be involved in the control of gene expression via regulating the transcription of target genes. In addition, in the process of clinical phenotype analysis, it was found that tRF-21-NB8PLML3E was negatively correlated with hypertrophy-related indicators such as left ventricular posterior wall thickness in diastole (LVPWD), and the correlation showed an increasing trend. It is exciting to consider tsRNA’s possible application in the treatment of cardiac hypertrophy in addition to its potential use as a biomarker for early detection and diagnosis of the condition [106].

### 7.2. Atrial Fibrillation

tRF is not only associated with cardiac hypertrophy but also participates in the regulation of a variety of CVDs. For example, tsRNA-5008a can promote the development of AF. Xie et al. [68] found that the level of tsRNA-5008a in HL-1 cells and the myocardial tissue increased after induction of Ang II, then aggravated the development of AF. Subsequently, researchers speculated that tsRNA-5008a may have the ability to regulate ferroptosis, and they found that ferroptosis might be markedly inhibited by tsRNA-5008a knockdown, which targets SLC7A11 (a ferroptosis-related indicator) through AGO2, thereby reducing AF in vivo and in vitro. This finding not only provides new evidence for the role of tRF in AF but also elaborates on the link between tRF and ferroptosis. This will help in the study of tRF, CVD, and CD.

### 7.3. Heart Failure

HF is a public health issue that is quickly expanding and currently affects more than 37.7 million people all over the world. HF is the early stage of heart function damage caused by many causes. HF is, to a large extent, a geriatric syndrome. The prevalence and incidence also tended to increase with age [107]. HF patients show many symptoms that influence the quality of life, including poor dyspnea, fatigue, and exercise tolerance [108]. In addition, the morphological structure of the heart also changes. The heart of patients with HF shows the accumulation and swelling of epicardial adipose tissue (EAT) (metabolically active visceral adipose tissue located in the atrioventricular sulcus and interventricular sulcus). EAT, as an active metabolic factor, can produce a variety of bioactive molecules, including small non-coding RNA particles. Researchers have thus hypothesized that sncRNA may have a regulatory role [109]. Currently, the role of tsRNAs in heart failure (HF) has also been demonstrated. Zhao et al. identified 343 tsRNA expression profiles in EAT and found dense target gene interactions between tRF-Tyr-GTA-010 and tRF-Tyr-GTA-011. These tsRNAs primarily target genes involved in calcium transport, sphingolipid and adrenergic signaling pathways, as well as the regulation of calcium homeostasis. Calcium transport controls intracellular calcium concentration, which plays a protective role in preventing the progression of heart failure [110]. In patients who have suffered an acute myocardial infarction (AMI) that is worsened by heart failure, sacubitril/valsartan has the ability to restore cardiac function. However, lack of access to treatment is referred to as sacubitril/valsartan resistance (SVR) [111]. One study compared the SVR and non-sacubitril/valsartan resistance (NSVR) tsRNA expression differences between patients; according to the receiver-operating characteristic curve (ROC) analysis, tRF-59:76-Tyr-GTA2-M3, tRF-60:76-Val-AAC-1-M5, and tRF-1:29-Gly-GCC-1 were the biomarkers of treatment heterogeneity. In addition, an analysis of their targeted mRNAs revealed their ability to regulate the transmembrane transport of calcium and sodium ions in cardiomyocytes and influence ion channels to regulate the contractile activity of cardiomyocytes. Therefore, these targets have the capacity to be used for therapeutic purposes in the treatment of HF [112]. These results explain tsRNA as a pathway to AMI with a control effect on HF.

### 7.4. Fulminant Myocarditis

Fulminant myocarditis (FM) is an infectious inflammatory disease. The mortality and fatality rates among children and women are high. It is characterized by severe disturbance of blood flow, leading to cardiogenic shock and arrhythmia, or multi-organ failure, leading to death. FM [113] is typically a diseases that directly induces myocardial cell injury and immune response-mediated tissue damage [114]. So far, there is reason to be optimistic about the potential of tRF as a diagnostic and therapeutic target for the treatment of FM. Patients diagnosed with juvenile FM were shown to have an elevated level of tiRNA-Gln-TTG-001 expression, and it has the potential to be a marker for the stage of FM. Further investigation revealed that the target gene of tiRNA-Gln-TTG-001 is associated with the metabolic processes of myotubes. Therefore, the signaling pathway of tRF involved in the regulation of myocardial cell metabolism has the potential to become a therapeutic pathway for FM [113].

### 7.5. Myocardial Ischemia/Reperfusion Injury

MI has long been one of the most challenging problems facing clinicians and researchers [115]. To treat this kind of situation, reperfusion is the preferred treatment in a timely manner [116]. Even so, the damage to the heart cannot be ignored. Thus, a 3′tRF (tRF HC83) derived from ginseng tRNAGln (UUG) was identified. It has a significant cardioprotective effect in vitro and in vivo and has a highly effective anti-ischemia/reperfusion injury effect. Mechanically, tRF HC83 directly binds to MIAT (myocardial infarction-associated transcript) to down-regulate its expression and leads to the subsequent up-regulation of vascular endothelial growth factor A (VEGFA) expression. In addition, HC83 also has the ability to maintain cytoskeletal integrity and mitochondrial function in cardiomyocytes, thereby improving cardiac function [117].

### 7.6. Atherosclerosis

Atherosclerosis (AS) is a chronic inflammatory disease, characterized by the accumulation of lipids, fibrous materials, and inflammatory cells within the arterial wall. The rupture of unstable atherosclerotic plaques, arterial stenosis, or thrombus formation leading to vessel occlusion can all trigger the onset of acute cardiovascular diseases [118]. Previous studies have established that aberrant proliferation and migration of vascular smooth muscle cells (VSMCs) are the primary etiological factors of atherosclerosis (AS). Studies have demonstrated that statins can modulate the expression of miRNAs, such as miR-139-3p, which significantly enhances cardiac repair capacity by promoting macrophage polarization [116]. The mechanism of statins involves the mevalonate pathway, primarily through the inhibition of HMG-CoA reductase to reduce cholesterol levels. The mevalonate pathway is implicated in various cellular metabolic and signaling processes, potentially indirectly influencing the biosynthesis and function of tsRNAs [119].

Recently, emerging evidence has indicated that tRNA-derived small RNAs (tsRNAs) possess the capability to modulate the state of VSMCs. Through small RNA sequencing, He et al. identified 315 differentially expressed tsRNAs in the AS group, with 131 up-regulated and 184 down-regulated [120]. Particularly, tRF-Gly-GCC-009 is significantly up-regulated and may participate in the pathogenesis of AS by modulating the expression of cell adhesion molecules (CAMs). With the development of bioinformatics technologies, researchers indicate that tRF-Gly-GCC-009 is associated with multiple signaling pathways, including the Apelin signaling pathway, Notch signaling pathway, calcium signaling pathway, and MAPK signaling pathway [121]. These findings suggest that tsRNAs hold great potential in controlling the progression of AS by regulating cholesterol metabolism, inflammatory responses, endothelial-mesenchymal transition, cellular stress, differentiation, and other aspects.

## 8. Discussion

Continuous discoveries of new tRFs have been made with the advancement and implementation of high-throughput sequencing technologies. Initially considered tRNA by-products, tRF has become a new class of sncRNAs with important biological functions. The biological functions and potential mechanisms of tRF are still under constant study, especially emphasizing the expression of tRF through special de novo RNA silencing, transcriptional repression, and translational regulation. These molecular mechanisms are essential for our understanding of the prevention and development of diseases.

Here, we review the origin of ncRNA, such as lncRNA, circRNA, and miRNA, as well as tsRNA, the focus of our study. tsRNA is not a random degradation product of tRNA but is generated by the cleavage of tRNA by a specific endonuclease. tsRNA is divided into tRNA halves and tRF depending on where it is cleaved. These tRFs include canonical fragments with 5′ or 3′ ends from mature tRNA, as well as non-canonical fragments such as tRF-1 and tiRNA. At the same time, the process of tRNA modification, which has an effect on the fate of tsRNA, is responsible for regulating the biogenesis of tsRNA. In addition, some tRFs such as tRF-3 have a similar way to miRNA to bind to the AGO protein to engage in the expression regulation of target genes, indicating the similarity between tsRNA and miRNA. When it comes to the process of biological activity, tRF is an extremely important component. It has been demonstrated that tRF uses a special mechanism involving NRS and contact with RBPs to take part in the expression control of target genes. It was also found that it may be involved in the regulation of reverse transcription, chromatin access, apoptosis, etc.

At present, it has been reported that tRF participates in the regulation of signaling pathways implicated in a range of disorders, including CVD, cancer, and so on. The expression of tsRNA in different diseases is different, and its function covers a diverse array of biological processes. We investigated the potential of tRF as a biomarker in different cancers such as BC, GC, and CRC. The mechanism by which tRF affects cancer development is described. This highlights the molecular efficacy of tRF to provide novel therapeutic approaches for cancer. Although the clinical value of tsRNA still needs to be further studied, there is increasing evidence that tsRNA has the potential to serve as a marker for the diagnosis and prognosis of CVDs. The abovementioned examples of tRF in cardiac hypertrophy and HF provide motivation for subsequent research on the regulation of tRF in CVDs. The presence of tRF in patient plasma has great potential to become an emerging therapeutic target.

The therapeutic value of tsRNA in the clinic is a promising area of research. As summarized in this review, tsRNA plays a role in controlling numerous diseases and has the potential to become a significant therapeutic tool in clinical practice. It is noteworthy that tsRNA quantity varies depending on the tissue and causes a wide range of bioactive effects. Therefore, controlling the expression activity of tsRNA is likely to be the primary focus of future research. To summarize, tsRNAs with various biological functions have been continuously discovered, and the current preliminary results reveal the association between tsRNAs and CVD, but the regulatory network of tsRNAs expression has still not been fully explored. In the future, there is a pressing requirement for the comprehensive study of the molecular mechanism to better understand tRF in CVDs, maximize the utilization of its potential as a medicinal target, and apply it to clinical practice as soon as possible.

## 9. Conclusions

Despite recent progress in understanding the roles of tsRNAs in cardiovascular diseases (CVDs), their regulatory networks remain underexplored. TsRNAs exhibit disease-specific expression profiles, positioning them as promising biomarkers and therapeutic targets in cancers (e.g., breast cancer [BC], colorectal cancer [CRC]) and CVDs (e.g., heart failure [HF]). However, translational challenges, including mechanistic ambiguity and clinical validation barriers, persist. Future research must prioritize elucidating tsRNA-driven molecular pathways, expediting preclinical validation, and translating these findings into targeted therapies. Addressing these gaps will unlock the potential of tsRNAs as precision tools for diagnosis and treatment, redefining therapeutic paradigms in precision medicine.

## Figures and Tables

**Table 1:** Comparison of tsRNA and miRNA biogenesis.

Subject	miRNA	tsRNA
Length	21–23 nt	tiRNA 30–40 nt tRF 14–30 nt
Processing of precursors	The transcription of pri-miRNA is mediated by RNApol II	The transcription of pre-tRNA is mediated by RNApol III
Modification of precursors	With a 3′ polyadenylation tail and a 5′ methylation cap, pri-miRNA is processed into pre-miRNA by the RNase III enzyme Drosha and its dsRNA domain	The 5′ leading sequence and the 3′ tail sequence and intron are cleaved, and attach to the 3′-end CCA sequence
Process of maturation	Dicer processes pre-miRNA into a duplex after exportation protein 5 (XPO5) exports it to the cytoplasm	tsRNA is cleaved at specific positions by different endonucleases
Mechanisms of function	miRNA combine with AGO proteins to form RISC, which can bind to the 3′ UTR of specific mRNA to destabilize mRNA and inhibit translation	tsRNA can interact with the AGO protein and cleave target mRNA to inhibit translation. Or it interacts with RBP to inhibit the binding of target genes to it
RNA modifications regulate biogenesis	N6-methyladenosine(m6A), 7-methylguanosine(m7G), Pseudouridine acidification(φ)	5-methylcytosine(m5C), Queuosine modification(Q)

**Table 2:** The modes of tsRNA participation in disease regulation.

tsRNA	Downstream Targets	Cell Death/ Signaling Pathways	Diseases
tRFs1	APAF1	Apoptosis	Cardiac hypertrophy
tRF3-Thr- AGT	Z-DNA	Pyroptosis	Acute pancreatitis
tRF-47-58ZZJQJYSWRYVMMV5BO		Pyroptosis	Non-alcoholic steatohepatitis
tRFHis-GTG	AGO2	Necrosis	Ischemic stroke
tRFLeu-CAG	AGO2	Necrosis	Ischemic stroke
tsRNA-5008a	SLC7A11	Ferroptosis	Atrial fibrillation
tRF-23-Q99P9P9NDD	ACADSB	Ferroptosis	Gastric cancer
tiRNA-Lys-CTT-003	GRSF1	Ferroptosis	Acute kidney injury
tsRNA-13502		Ferroptosis	Non-small cell carcinoma
tRNA-Cys-GCA	VAV2	Autophagy	Glioma
tRF-64-85-Leu-AAG-M4	Rnf6	Autophagy	Traumatic brain injury
tsRNA-5006c	M5006	Hippo/MAPK/Wnt /Ras	Calcific aortic valve disease
5′-tRF-GlyGCC	ALKBH3		Colorectal cancer
tRF-3019a	FBXO47		Gastric cancer
tRF-17-79MP9PP	THBS1	FZD3/Wnt/β-Catenin	Breast cancer
tRF-3017A	NELL2		Gastric cancer
tRF-24-V29K9UV3IU	GPR78	Apoptosis	Gastric cancer
tRF3E	NCL		Breast cancer
AS-tDR-007333	HSPB1		Non-small cell carcinoma
5′-tiRNA-Cys-GCA	STAT4	Apoptosis	Cardiac hypertrophy
tRF-60:76-Val-AAC-1-M5	Tnfrsf10b/Bcl2l1	Lipid/Ras/NF-κB	Atherosclerosis
tiRNA-Gln-TTG-001		Ras/MAPK/ PI001K-Akt	Fulminant myocarditis
tRF HC83	MIAT	Apoptosis	Myocardial ischemia/reperfusion

## Data Availability

Not applicable.

## Abbreviations

The following abbreviations are used in this manuscript:

aaRS	aminoacyl-tRNA synthetases
AF	Atrial fibrillation
AGO	Argonaute
AlkB3	AlkB homolog 3
AMO	anti-miRNA oligonucleotides
AMI	Acute myocardial infarction
ANG	Angiopoietin
AP	Acute pancreatitis
APAF1	Apoptotic protease-activating factor 1
BC	Breast cancer
CA	Carbohydrate antigen
CD	cell death
CEA	Carcinoembryonic antigen
CircRNA	Circular RNA
CLASH	Cross linking-ligation and sequencing of hybrids
CRC	Colorectal cancer
CVD	cardiovascular disease
Cytc	Cytochrome c
DNMT2	DNA methyltransferase 2
EAT	Epicardial adipose tissue
ELAC2 (RNase Z)	Endonuclease Z
ENCODE	Encyclopedia of DNA Elements
EZH2	Enhancer of zeste homolog 2
EF	elongation factor
FM	Fulminant myocarditis
GC	Gastric cancer
GO	Gene Ontology
HF	Heart failure
HSPB	Heat shock protein
KEGG	Kyoto Encyclopedia of Genes and Genomes
LNA	Locked nucleic acid
LncRNA	Long non-coding RNA
LVPWD	Left ventricular posterior wall thickness in diastole
MEF	mouse embryonic fibroblast
MiRNA	MicroRNA
m1A	N1-methyladenosine
m5C	5-methylcytosine
MI	Myocardial infarction
MIAT	Myocardial infarction associated transcript
Nt	Nucleotide
ncRNA	Non-coding RNA
NGS	Next-generation sequencing
NRS	New RNA silencing mechanism
NSCLC	Non-small cell lung cancer
NSUN2	NOP2/Sun RNA methyltransferase 2
NSVR	Non-sacubitril/valsartan resistance
PiRNA	Piwi-interactingRNA
PNKP	polynucleotide kinase phosphatase
Pre-miRNA	Precursor microRNA
Pri-miRNA	Primary microRNA
Pre-tRNA	Precursor tRNA
PTGS	Post-transcribed gene silencing
PUS7	Pseudouridine synthetase
PAR-CLIP	Photoactivatable-ribonucleoside-enhanced crosslinking and immunoprecipitation
Q	Queuosine
RASSF9	Ras association domain family member 9
RBP	RNA-binding protein
RIPK3	Receptor-dependent serine-threonine kinase 3
RISC	RNA-induced silencing complex
RNA-Seq	RNA sequencing
RNApol III	RNA polymerase III
ROC	Receiver operating characteristic curve
shRNA	Short hairpin RNA
sncRNA	Small non-coding RNA
siRNA	Small interfering RNA
sitRNA	Stress-induced tRNA-derived RNA
STAT4	Signal transducer and activator of transcription4
SVR	Sacubitril/valsartan resistance
TCGA	The Cancer Genome Atlas
tRNA	Transfer RNA
tRF	tRNA-derived fragment
tsRNA	tRNA-derived small RNA
tiRNA (tRH)	tRNA-derived stress-induced RNA
TGS	Transcriptional gene silencing
TOG	5′ terminal oligopurines
UTR	non-translational regions
VSMC	Vascular smooth muscle cell
VEGF	Vascular endothelial growth factor
YBX1	Y box binding protein 1
ZBP1	Z-DNA-binding protein
Ψ	Pseudouridine
